# Association of the HLA-DRB1 with Scleroderma in Chinese Population

**DOI:** 10.1371/journal.pone.0106939

**Published:** 2014-09-03

**Authors:** Dongyi He, Jiucun Wang, Lin Yi, Xinjian Guo, Shicheng Guo, Gang Guo, Wenzhen Tu, Wenyu Wu, Li Yang, Rong Xiao, Yuan Li, Haiyan Chu, Syeling Lai, Li Jin, Hejian Zou, John D. Reveille, Shervin Assassi, Maureen D. Mayes, Xiaodong Zhou

**Affiliations:** 1 Division of Rheumatology and Clinical Immunogenetics, the University of Texas Medical School at Houston, Houston, TX, United States of America; 2 Institute of Arthritis Research, Shanghai Academy of Chinese Medical Sciences, Guanghua Integrative Medicine Hospital, Shanghai, China; 3 Ministry of Education (MOE) Key Laboratory of Contemporary Anthropology and State Key Laboratory of Genetic Engineering, School of Life Sciences, Fudan University, Shanghai, China; 4 Institute of Rheumatology, Immunology and Allergy, Fudan University, Shanghai, China; 5 Gansu College of Traditional Chinese Medicine, Lanzhou, Gansu, China; 6 Yiling Hospital, Shijiazhuang, Hebei Province, China; 7 Shanghai Traditional Chinese Medicine-Integrated Hospital, Shanghai, China; 8 Division of Dermatology, Huashan Hospital, Fudan University, Shanghai, China; 9 Division of Rheumatology, Teaching Hospital of Chengdu University of TCM, Chengdu, Sichuan Province, China; 10 Department of Dermatology, Second Xiangya Hospital, Central South University, Changsha, Hunan Province, China; 11 Department of pathology, Baylor College of Medicine, Michael E. DeBakey VA Medical Center, Houston, TX, United States of America; 12 Division of Rheumatology, Huashan Hospital, Fudan University, Shanghai, China; Keio University School of Medicine, Japan

## Abstract

Multiple alleles of the Human leukocyte antigen (HLA) DRB1 have been strongly associated with systemic sclerosis (SSc) and its clinical or serological subsets. However, the associations vary in different ethnic populations. To define SSc-risk and/or -protective alleles of HLA-DRB1 in Chinese population, we studied a Han Chinese cohort containing 585 patients with SSc and 458 gender-matched, unrelated controls. The HLA-DRB1 genotyping was performed with sequence-based typing method. Exact *p*-values were obtained (Fisher’s test) from 2×2 tables of allele frequency and disease status. The major SSc-risk allele subtypes of HLA-DRB1 are the DRB1*15∶02 and *16∶02 in this Chinese cohort. Particularly, DRB1*15∶02 was most significantly associated with anti-centromere autoantibodies (ACA) positive, and DRB1*16∶02 with anti-topoisomerase I autoantibodies (ATA) positive patients. On the other hand, DRB1*01∶01 and *04∶06 were strong SSc-protective alleles in Chinese, especially in patients who were ACA positive and had diffuse cutaneous SSc (dcSSc), respectively. In addition, DRB1*11 and *07∶01 also showed significant association with SSc as a risk for and protection from SSc, respectively, and which is consistent with the studies of Spanish, US Caucasian and Hispanic populations. DRB1*15 was associated with ATA positive Chinese SSc that is consistent with Black South African and Korean SSc. These findings of HLA-DRB1 alleles in association with Chinese SSc provide the growing knowledge of genetics of SSc, and indicate that the genetic heterogeneity among ethnicities may significantly impact the complex trait of SSc.

## Introduction

Systemic sclerosis (SSc) is an immune-mediated disease of unknown etiology. It is characterized by fibrosis of skin and internal organs, and the presence of a group of mutually exclusive autoantibodies [1]. The most common SSc autoantibodies are anti-topoisomerase I autoantibody (ATA), anti-centromere autoantibody (ACA), and anti-RNA polymerase III autoantibody (RNAP3) [2], [3]. In addition, based on the extent of skin fibrosis, SSc can be classified into two clinical subsets: limited cutaneous (lcSSc) and diffuse cutaneous (dcSSc) SSc [4]. The latter subset is characterized by more rapid progression of skin and visceral involvement, as well as poorer prognosis [1], [4].

Although the etiology of SSc is still unknown, genetic factors clearly play an important role. There have been numerous genetic studies in SSc [5]–[17], including three genome-wide association studies (GWAS) [7]–[10] and one immunochip study [11], which demonstrated that the strongest genetic associations with SSc fell within the human leucocyte antigen (HLA) class II region. Specific HLA class II alleles were associated with SSc and its subtypes in multiple reports [7]–[18]. However, major SSc-associated HLA-DRB alleles vary in different ethnic populations.

In a multi-ethnic US study [12], DRB1*11, particularly DRB1*11∶04 was strongly associated with the risk of SSc, while DRB1*07∶01 was decreased (protective) in SSc in the US Caucasians and Hispanic individuals. In addition, HLA-DRB1*01 (particularly DRB1*01∶01) was associated with increased risk in patients who were ACA positive, while DRB1*11∶04 was a major risk factor for ATA positive SSc. However, these observations were not the same for SSc in African Americans [12], in whom DRB1*08∶01 was found to be significantly associated with SSc in the overall group of patients and most strongly associated in especially ATA positive group [12].

An independent study of a Spanish population supported the associations of HLA-DRB1*11 and DRB1*07∶01 with SSc [13]. In a study of UK population [14], the associations between HLA-DRB1*11 and SSc with ATA was concordant, but DRB1*04 and DRB1*08 were associated with ACA. The meta-analysis of Italian and Spanish SSc showed an association between HLA-DRB1*01 alleles and ACA [15]. In Black South Africans [16], DRB1*11 was associated with pulmonary fibrosis, ATA was associated with DRB1*15, and DRB1*0301 with the lcSSc. In a Korean SSc cohort [17], HLA-DRB1*15 was associated with ATA positive SSc. However, a Japanese population study did not show differences of frequencies of DRB1 alleles between SSc patients and healthy controls for the disease overall but did find differences for the autoantibody subsets [18].

These controversial results highlight the complexity and heterogeneity of HLA-DRB1 gene in different ethnic populations, and emphasize the importance of studying genetic associations of SSc within specific geographic distribution and racial/ethnic groups.

Chinese SSc patients have unique serological and clinical features with higher frequency of ATA, dcSSc and pulmonary fibrosis (PF) and lower anti-RNAP3 antibody frequency than their European-ancestry counterparts [19]. Associations between the HLA-DRB1 alleles and SSc have not been previously reported in a Chinese SSc population. Recently, we established an SSc cohort of Han Chinese through a multicenter SSc consortium in China as part of the International Network of Scleroderma Clinical Care and Research (InSCAR) program (http://www.inscar-global.org). The goal of this current study is to investigate the HLA-DRB1 alleles in association with potential risk for or protection from SSc in Han Chinese.

## Materials and Methods

### Patient enrollment

SSc patients of Han population were recruited in a multicenter study including hospitals and outpatient clinics in Shanghai, Hebei province, Sichuan province, and Hunan province in China. All patients met the American College of Rheumatology (ACR) classification criteria for SSc [20], except 3 patients were diagnosed with at least 3 out of 5 CREST features (Calcinosis, Raynaud’s phenomenon, Esophageal dysmotility, Sclerodactyly, and Telangiectasia) with sclerodactyly being mandatory [4]. A total of 585 patients with SSc and 458 gender-matched unrelated controls of Han population were examined in the studies. None of the controls had autoimmune disease. The studies were approved by the institutional review boards of the University of Texas Health Science Center at Houston, United States of America and Fudan University, Shanghai, China. Written informed consent was obtained from each study subject.

### Autoantibody Testing

Patient’s sera were tested for antinuclear antibodies (ANA) by indirect immunofluorescence using HEp-2 cells as antigen substrate (Antibodies, Davis, CA). ATA was detected by passive immunodiffusion against calf thymus extracts (INOVA, Diagnosis). ACA was determined by the pattern of staining on indirect immunofluorescence using HEp-2 cells. Anti-RNAP3 was detected utilizing commercially available kits (NBL, Nagoya, Japan).

### HLA-DRB1 genotying

Genomic DNA was extracted from peripheral blood cells from subjects. The HLA-DRB1 genotyping was performed with a sequence-based typing (SBT) method using SeCore Kits (Life Technologies, USA). Briefly, the allele-specific polymerase chain reactions (PCR) were performed using primers supplied in the SeCore kits, followed by sequencing exon 2 and 3 of the HLA-DRB1 gene, as well as an additional targeted sequencing on codon 86. The HLA SBT uTYPE 6.0 program (Life Technologies) was used in sequencing analysis and assigning HLA-DRB1 alleles.

### Statistical analysis

Exact *p* values were obtained (Fisher’s test) from 2×2 tables of allele counts and disease status. The *p* values less than 0.05 were considered nominal significance. We applied both nominal significance and a strict “Bonferroni” correction to the *p* values, which allows the readers to consider the context, due to extensive and long-range haplotypes in the HLA-DRB1 region, a Bonferroni correction is considered highly conservative.

Permutation analysis (10,000 times) was conducted to validate previous chi-square test.

## Results

Autoantibody tests showed that 92% of SSc patients were ANA positive. There were 460 patients examined for ATA with 214 being positive (46.5%); 440 were examined for ACA with 92 positive (20.7%); 365 were examined for anti-RNAP3 with 7 positive (1.9%). Out of 433 patients who were examined with chest X-ray and/or CT, 295 were diagnosed as pulmonary fibrosis (68%).

A total of thirteen DRB1 alleles (two-digit typing) were found in cases and controls in this Chinese cohort, and the common ones of control group are DRB1*09 (15.7%), *15 (13.8%), *04 (13.5%), *12 (13%) and *07 (11.1%). The associations of these alleles with SSc are displayed in Table 1. Bonferroni correction for significance was calculated as *P*<0.0038. According to this correction, the HLA-DRB1*01 was significantly decreased, while DRB1*03, *11, *15 and *16 were significantly increased in SSc patients (Table 1). In studies of SSc subsets, DRB1*11 was more significantly associated with dcSSc, while DRB1*15 and *16 were more significantly associated with SSc patients who were ATA positive and dcSSc (Table 2). In addition, DRB1*16 and *03 were significantly increased in SSc patient with pulmonary fibrosis, and DRB1*10 was associated with SSc patients with ACA (Table 2).

**Table 1:** Distribution of major HLA-DRB1 alleles in Chinese SSc patients and controls.

Alleles	SSc	%	control	%	*p	OR (95% CI)
**DRB1** * **01**	**25**	**2.1**	**43**	**4.7**	**0.0011**	**0.44 (0.27–0.73)**
**DRB1** * **03**	**51**	**4.4**	**18**	**2.0**	**0.0024**	**2.27 (1.32–3.91)**
DRB1*04	123	10.5	124	13.5	0.034	0.75 (0.58–0.98)
DRB1*07	88	7.5	102	11.1	0.0044	0.65 (0.48–0.88)
DRB1*08	114	9.7	72	7.9	0.13	1.27–0.93)
DRB1*09	153	13.1	144	15.7	0.09	0.81 (0.63–1.03)
DRB1*10	19	1.6	13	1.4	0.71	1.15 (0.56–2.33)
**DRB1** * **11**	**90**	**7.7**	**40**	**4.4**	**0.0018**	**1.83 (1.24–2.68)**
DRB1*12	128	10.9	119	13.0	0.15	0.82 (0.63–1.07)
DRB1*13	64	5.5	59	6.4	0.31	0.83 (0.57–1.19)
DRB1*14	50	4.3	50	5.5	0.21	0.77 (0.52–1.16)
**DRB1** * **15**	**224**	**19.1**	**126**	**13.8**	**0.0011**	**1.49 (1.17–1.88)**
**DRB1** * **16**	**41**	**3.5**	**6**	**0.7**	**1.4×10^−5^**	**5.51 (2.23–14.5)**
**total**	**1170**		**916**			

*nominal significance level: P<0.05; Bonferroni correction for significance was calculated as P<0.0038.

**Table 2:** Two-digit allele frequencies of HLA-DRB1 between SSc subsets and controls.

	Cont (%)	ATA (+) (%)	p	OR (95% CI)
DRB1*11	4.4	8.1	0.0056	1.92 (1.2–3.1)
**DRB1** * **15**	**13.8**	**21.2**	**0.00052**	**1.69 (1.25–2.27)**
DRB1*15∶01	10.6	11.3	0.7	1.1 (0.73–1.57)
** DRB1** * **15∶02**	**2.1**	**6.5**	**4.2×10^−5^**	**3.3 (1.7–6.1)**
**DRB1** * **16**	**0.7**	**4.1**	**7×10^−6^**	**6.6 (2.6–16.7)**

	Cont (%)	ACA (+) (%)	p	OR (95% CI)
**DRB1** * **10**	**1.4**	**6.7**	**0.00022**	**5.01 (1.95–12.9)**
DRB1*11	4.4	10.6	0.0059	2.59 (1.29–5.22)
DRB1*15	13.8	20	0.08	0.6 (0.95–2.7)
DRB1*15∶01	10.6	5.8	0.12	0.52 (0.2–1.3)
** DRB1** * **15∶02**	2.1	13.5	<10^−7^	7.3 (3.4–16)

	Cont (%)	SSc with PF (%)	p	OR (95% CI)
**DRB1** * **03**	**2.0**	**4.7**	**0.0026**	**2.47 (1.35–4.52)**
DRB1*15	13.8	19.4	0.004	1.51 (0.14–2)
DRB1*15∶01	10.6	10.7	0.97	1 (0.71–1.43)
** DRB1** * **15∶02**	**2.1**	**6.8**	**4.4×10^−6^**	**3.5 (1.9–6.3)**
**DRB1** * **16**	**0.7**	**3.3**	**0.0001**	**5.2 (2.1–13.2)**

	Cont (%)	dcSSc (%)	p	OR (95% CI)
DRB1*03	2.0	4.5	0.0073	2.3 (1.2–4.4)
**DRB1** * **11**	**4.4**	**8.7**	**0.00096**	**2.09 (1.3–3.3)**
**DRB1** * **15**	**13.8**	**21.7**	**0.00014**	**1.7 (1.3–2.3)**
DRB1*15∶01	10.6	11.4	0.64	1.1 (0.76–1.5)
** DRB1** * **15∶02**	**2.1**	**7.3**	**1×10^−6^**	**3.7 (2.1–6.8)**
**DRB1** * **16**	**0.7**	**3.6**	**3.6×10^−5^**	**5.7 (1.3–14.5)**

	Cont (%)	lcSSc (%)	p	OR (95% CI)
DRB1*01	4.7	1.5	0.004	0.3 (0.13–0.71)
DRB1*07	11.1	6.3	0.0063	0.54 (0.35–0.85)
**DRB1** * **16**	**0.7**	**2.9**	**0.00095**	**4.6 (1.7–12.3)**

*nominal significance level: P<0.05; Bonferroni correction for significance was calculated as P<0.0038. For DRB1*15, two major subtypes also were displayed.

A total of twenty DRB1 allele subtypes (four-digit typing) were found with frequency equal or larger than 1% in the cases and/or controls. The common ones of control group are DRB1*09∶01 (15.7%), *07∶01 (11.1%), *15∶01 (10.6%) (Table 3). The associations of these allele subtypes with SSc are displayed in Table 3. Bonferroni correction for significance was calculated as P<0.0025. According to the correction, the HLA-DRB1*01∶01, *04∶06, *07∶01 and *12∶02 were significantly decreased in SSc, while DRB1*15∶02 and *16∶02 were strong risk alleles for SSc (Table 3). In analysis of SSc subsets with the HLA-DRB1 allele subtypes (Table 4), DRB1*01∶01 and DRB1*04∶06 were significantly decreased and increased, respectively, in SSc patients who were ACA negative, lcSSc or ATA positive; DRB1*07∶01 and *08∶02 were associated with lcSSc with decreased and increased occurrence, respectively; DRB1*12∶02 was increased in SSc patients with ATA or pulmonary fibrosis; DRB1*15∶02 and *16∶02 represented the major risk alleles for ACA positive and ATA positive SSc, respectively.

**Table 3:** Distribution of major HLA-DRB1 allele subtypes in Chinese SSc patients and controls.

Alleles	SSc	%	control	%	p-values^a^	p-values^b^	OR	95% CI
**DRB1** * **01∶01**	**17**	**1.50%**	**43**	**4.70%**	**1×10^−5^**	<10^−4^	**0.3**	**0.16–0.54**
**DRB1** * **03∶01**	**47**	**4.00%**	**17**	**1.90%**	**0.0045**	**0.0024**	**2.21**	**1.25–4.04**
DRB1*04∶03	19	1.60%	14	1.50%	0.86	0.8564	1.06	0.51–2.25
DRB1*04∶05	42	3.60%	51	5.60%	0.03	0.0241	0.63	0.41–0.98
**DRB1** * **04∶06**	**20**	**1.70%**	**39**	**4.30%**	**4.9×10^−4^**	**0.0001**	**0.39**	**0.22–0.7**
**DRB1** * **07∶01**	**85**	**7.30%**	**102**	**11.10%**	**0.0021**	**0.0021**	**0.63**	**0.46–0.86**
DRB1*08∶02	22	1.90%	4	0.40%	0.0032	0.0031	4.37	1.42–15
DRB1*08∶03	72	6.20%	62	6.80%	0.57	0.5277	0.9	0.63–1.30
DRB1*09∶01	147	12.60%	144	15.70%	0.039	0.0298	0.77	0.6–0.99
DRB1*10∶01	18	1.50%	13	1.40%	0.82	0.7157	1.09	0.50–2.36
DRB1*11∶01	68	5.80%	36	3.90%	0.05	0.0459	1.51	1.00–2.19
DRB1*12∶01	55	4.70%	39	4.30%	0.63	0.5984	1.11	0.71–1.72
**DRB1** * **12∶02**	**59**	**5.00%**	**78**	**8.50%**	**0.001**	**0.0014**	**0.57**	**0.4–0.82**
DRB1*13∶01	12	1.00%	19	2.10%	0.05	0.0436	0.49	0.22–1.06
DRB1*13∶02	24	2.10%	35	3.80%	0.016	0.0121	0.53	0.30–0.92
DRB1*14∶01	13	1.10%	15	1.60%	0.3	0.2515	0.67	0.30–1.51
DRB1*14∶05	20	1.70%	15	1.60%	0.9	0.8676	1.04	0.51–2.16
DRB1*15∶01	131	11.20%	97	10.60%	0.66	0.6322	1.06	0.80–1.42
**DRB1** * **15∶02**	**69**	**5.90%**	**19**	**2.10%**	**1.6×10^−5^**	**<10^−4^**	**2.96**	**1.72–5.13**
**DRB1** * **16∶02**	**41**	**3.50%**	**6**	**0.70%**	**1.4×10^−5^**	**<10^−4^**	**5.51**	**2.23–14.48**
**Others** *	**189**	**16.2%**	**68**	**7.4%**				
**Total**	**1170**		**916**					

p-values^a^ were derived from chi-square test.

p-values^b^ were derived from 10,000 times permutation test.

*nominal significance level: P<0.05; Bonferroni correction for significance was calculated as P<0.0025; Others indicate minor alleles.

**Table 4:** Comparisons between SSc subsets and controls.

		lcSSc	dcSSc	ATA (+)	ATA (−)	ACA (+)	ACA (−)	PF (+)	PF (−)
DRB1*01∶01	p	0.00027	0.0077	0.002	0.005	0.96	9.1×10^−6^	0.0028	0.016
	OR	0.2	0.4	0.3	0.4	1	0.2	0.4	0.3
	(95% CI)	(0.04–0.5)	(0.2–0.8)	(0.1–0.7)	(0.2–0.8)	(0.4–2.8)	(0.1–0.4)	(0.2–0.8)	(0.1–0.9)
DRB1*03∶01	p	0.027	0.035	0.28	0.003	0.96	0.06	0.012	0.47
	OR	2.2	2	1.5	2.5	1	1.8	2.2	1.4
	(95% CI)	(1.0–4.5)	(1–4.1)	(0.7–3.4)	(1.3–5)	(0.2–4.4)	(0.9–3.6)	(1.1–4.4)	(0.5–3.6)
DRB1*04∶06	p	0.063	0.0002	0.001	0.007	0.25	0.001	0.0082	0.005
	OR	0.5	0.2	0.2	0.4	0.4	0.3	0.4	0.2
	(95% CI)	(0.2–1.1)	(0.1–0.5)	(0.1–0.6)	(0.2–0.8)	(0.1–1.9)	(0.2–0.7)	(0.2–0.8)	(0.03–0.7)
DRB1*07∶01	p	0.0024	0.178	0.0099	0.12	0.28	0.018	0.02	0.19
	OR	0.5	0.8	0.6	0.7	0.7	0.7	0.6	0.7
	(95% CI)	(0.3–0.8)	(0.5–1.1)	(0.4–0.9)	(0.5–1.1)	(0.7–1.5)	(0.5–0.9)	(0.4–0.9)	(0.4–1.2)
DRB1*08∶02	p	0.00035	0.057	0.01	0.051	0.004	0.005	0.0025	0.069
	OR	6.3	3.1	4.3	3.2	6.8	4.3	4.9	3.4
	(95% CI)	(1.8–23.5)	(0.8–12.7)	(1.2–17)	(0.8–13)	(1.2–36.3)	(1.3–15.8)	(1.5–18)	(0.7–16.1)
DRB1*12∶02	p	0.0193	0.021	0.0006	0.04	0.0063	0.014	0.001	0.064
	OR	0.6	0.6	0.4	0.6	0.1	0.6	0.5	0.6
	(95% CI)	(0.3–0.9)	(0.4–1)	(0.2–0.7)	(0.4–1.0)	(0.01–0.7)	(0.4–0.9)	(0.3–0.8)	(0.3–1.1)
DRB1*15∶02	p	0.00138	1×10^−6^	4.2×10^−5^	3.6×10^−5^	<10^−7^	1.3×10^−5^	4.4×10^−6^	0.00046
	OR	2.7	3.7	3.3	3.2	7.3	3.2	3.5	3.1
	(95% CI)	(1.4–5.2)	(2.1–6.8)	(1.7–6.1)	(1.7–5.9)	(3.4–16)	(1.8–5.7)	(1.9–6.3)	(1.5–6.4)
DRB1*16∶02	p	0.00096	6×10^−5^	5.8×10^−6^	0.006	0.72	1.7×10^−6^	0.0001	0.00062
	OR	4.6	5.5	6.6	3.7	1.47	6.6	5.2	5.2
	(95% CI)	(1.6–13.8)	(2.0–15.5)	(2.4–18.6)	(1.3–11.1)	(0.2–12.1)	(2.6–17.8)	(2–14.6)	(1.7–16.4)

*nominal significance level: P<0.05; Bonferroni correction for significance was calculated as P<0.0025.

Comparisons between dcSSc and lcSSc, autoantibody positive and negative SSc, and SSc with and without PF, only DRB1*01∶01 and *15∶02 were observed to be significantly increased in ACA positive SSc compared to ACA negative SSc patients. It is important to know that this comparison may be lack of statistic power for some alleles.

HLA-DRB1*11∶04, an uncommon allele in the Chinese cohort, was increased in SSc (0.77% in cases vs. 0.22% in controls), but its p value is 0.085 with odds ratio (OR) 3.5.

We were underpowered to perform meaningful association studies in the anti-RNAP3 positive subgroup because the frequency of this antibody was only 1.9% (7 cases) in this cohort.

It is worth noting that Bonferroni correction is extremely conservative. It can lead to false negative errors of unacceptable levels. Therefore, we reported the nominal p-values (p<0.05) in each table.

The permutation test showed same significant pattern with traditional chi-square test. DRB1*01∶01, DRB1*15∶02, DRB1*16∶02 were significantly associated with SSc at p-values <10^−4^, while DRB1*04∶06, DRB1*12∶02 and DRB1*07∶01 were strongly associated with SSc achieving the p-values of 0.0001, 0.0014 and 0.0021, respectively (Table 3). DRB1*03∶01 showed a significant association with SSc, while it is not significant in traditional chi-square test with “Bonferroni” correction.

As we previously reported that DQB1*05∶01 was strongly associated with ACA positive SSc in Han Chinese [21]. In fact, DRB1*15∶02/DQB1*05∶01 was considered as a haplotype in Asia population [22]. Other alleles of the DQB1 did not show significant impact on those DRB1 alleles observed in association with SSc.

## Discussion

Human HLA genes are extremely polymorphic. Specific alleles of the HLA genes determined by complex gene sequence polymorphisms encode amino acid sequences with distinct affinity for antigenic peptides (epitopes) for effective immune surveillance. In many aspects of the immune response, a large variety of HLA alleles is considered necessary to protect the human body. However, some of these alleles also confer susceptibility to a variety of autoimmune or immune-mediated diseases, e.g. SSc [7]–[18], rheumatoid arthritis (RA) [23], ankylosing spondylitis [24] and type 1 diabetes [25].

According to SSc GWAS, the HLA genes confer the major susceptibility to SSc [7]–[10]. Several studies indicated that the HLA-DRB1 gene in particular contains major SSc-risk and -protective alleles. However, SSc-associated HLA-DRB1 alleles appeared only partially concordant in different ethnic populations [12]–[18]. Studies of a Chinese cohort herein supported this notion, and a comparison of SSc-associated HLA-DRB1 alleles of the Chinese cohort with the reports of other ethnic populations is displayed in Table 5. In particular, significantly increased HLA-DRB1*11 in SSc in the Han Chinese is concordant with the reports of studies in US Caucasian and Hispanics [12], Spanish [13], UK [14] and Black South African populations [16]. A strong association of DRB1*15 with SSc is consistent in the Chinese Han, Black South Africans [17], Koreans [18] and Thai patients [26], which suggests it is specific to populations of Asian and South African. On the other hand, the associations between DRB1*04 and *08 with ACA positive SSc reported in UK studies [14] were not observed in this Chinese cohort, which instead showed an association of DRB1*10 with ACA positive cases. In addition, it is worth noting that DRB1*15 and *16 were strongly associated with PF along with dcSSc and ATA (+) in Chinese SSc patients (Table 2). Interestingly, these two alleles also were reported in association with interstitial lung disease (ILD) in Japanese RA patients [27].

**Table 5:** Comparison of SSc-associated HLA-DRB1 alleles of Chinese cohort with the reports of other ethnic populations (*UP: under power).

	US Caucasian	US Hispanics	US African American	Spanish	UK	SouthAfricans	Korean	Thai	Chinese Han
↑DRB1*01∶01 in ACA (+)	yes	yes		yes					no
↑DRB1*11∶04 in ATA (+)	yes	yes			yes				*UP
↑DRB1*08∶01 in ATA			yes						no
↑DRB1*0301 in lcSSc						yes			yes
↓DRB1*07∶01	yes	yes		yes					yes
↑DRB1*04 in ACA					yes				no
↑DRB1*08 in ACA					yes				no
↑DRB1*11 in SSc	yes	yes		yes	yes	yes			yes
↑DRB1*15 in ATA						yes	yes	yes	yes

Analysis of HLA-DRB1 allele subtypes showed that DRB1*07∶01 was consistently protective from SSc in Han Chinese, US Caucasian, US Hispanic and Spanish patients [12], [13]. A decreased DRB1*03∶01 in lcSSc was concordant between Han Chinese (at nominal significance level) and Black South Africans [16]. DRB1*11∶04, a major risk allele for ATA positive SSc in US Caucasian [12], US Hispanic [12] and UK populations [14], is a rare allele in Han Chinese. Although it was increased (0.77% in cases vs. 0.22% in controls) in Chinese SSc, the association was not significant likely due to small numbers and a lack of statistic power.

The major SSc-risk allele subtypes of HLA-DRB1 are the DRB1*15∶02 and *16∶02 in this Chinese cohort. Particularly, DRB1*15∶02 was most significantly associated with ACA positive, and DRB1*16∶02 with ATA positive and ACA negative Chinese SSc patients. On the other hand, DRB1*01∶01 and *04∶06 were strong SSc-protective alleles in Chinese, especially in patients who were ACA positive and had dcSSc, respectively. In contrast, the studies of US Caucasian, US Hispanic and UK SSc cohorts indicated that DRB1*01∶01 was a risk allele for ACA positive SSc [12], [14]. In addition, a previously reported association between DRB1*08∶01 and ATA positive SSc in African Americans [12] was not observed in the Chinese cohort.

While examining HLA region by imputing through genome-wide SNP data, Raychaudhuri and others found SNP variants corresponding to five amino acids including three in HLA-DRβ1 and one in each HLA-B (at position 9) and HLA-DPβ1 almost completely explain the MHC association to seropositive RA risk [28]. To examine whether identified SSc-risk alleles from different ethnic populations share any sequence variants or corresponding amino acids different from non-risk alleles of SSc, each sequence of these SSc-risk alleles was compared with each other and with other non-risk alleles. Such a unique sequence variant or an encoded amino acid was not fund among these SSc-risk alleles.

In summary, this is the first report of HLA-DRB1 studies in Chinese SSc. Compared to the results of previously reported studies in several other ethnic populations, DRB1*11 and *07∶01 showed the most consistent association with SSc as a risk for and protection from SSc, respectively. Identified associations of some specific HLA-DRB1 alleles with Chinese SSc were not reported in, or discordant from the studies of other ethnic populations, which need to be confirmed in a large sample size of SSc cohort and/or other ethnic populations. In fact, previous studies of different populations already demonstrated ethnic differences in genetic associations with SSc. Therefore, genetic heterogeneity among ethnicities may significantly impact the complex trait of SSc.
